# Distorted Coarse Axon Targeting and Reduced Dendrite Connectivity Underlie Dysosmia after Olfactory Axon Injury

**DOI:** 10.1523/ENEURO.0242-16.2016

**Published:** 2016-10-17

**Authors:** Aya Murai, Ryo Iwata, Satoshi Fujimoto, Shuhei Aihara, Akio Tsuboi, Yuko Muroyama, Tetsuichiro Saito, Kazunori Nishizaki, Takeshi Imai

**Affiliations:** 1 Laboratory for Sensory Circuit Formation, RIKEN Center for Developmental Biology, Chuo-ku 650-0047, Japan; 2 Department of Otolaryngology–Head and Neck Surgery, Okayama University Graduate School of Medicine, Dentistry and Pharmaceutical Sciences, Okayama 700-8558, Japan; 3 Laboratory for Molecular Biology of Neural System, Advanced Medical Research Center, Nara Medical University School of Medicine, Kashihara 634-8521, Nara, Japan; 4 Department of Developmental Biology, Graduate School of Medicine, Chiba University, Chiba 260-8670, Japan; 5 Graduate School of Biostudies, Kyoto University, Kyoto 606-8501, Japan

**Keywords:** dysosmia, glomerular map, olfactory bulb, olfactory sensory neurons, regeneration, traumatic axon injury

## Abstract

The glomerular map in the olfactory bulb (OB) is the basis for odor recognition. Once established during development, the glomerular map is stably maintained throughout the life of an animal despite the continuous turnover of olfactory sensory neurons (OSNs). However, traumatic damage to OSN axons in the adult often leads to dysosmia, a qualitative and quantitative change in olfaction in humans. A mouse model of dysosmia has previously indicated that there is an altered glomerular map in the OB after the OSN axon injury; however, the underlying mechanisms that cause the map distortion remain unknown. In this study, we examined how the glomerular map is disturbed and how the odor information processing in the OB is affected in the dysosmia model mice. We found that the anterior–posterior coarse targeting of OSN axons is disrupted after OSN axon injury, while the local axon sorting mechanisms remained. We also found that the connectivity of mitral/tufted cell dendrites is reduced after injury, leading to attenuated odor responses in mitral/tufted cells. These results suggest that existing OSN axons are an essential scaffold for maintaining the integrity of the olfactory circuit, both OSN axons and mitral/tufted cell dendrites, in the adult.

## Visual Abstract

**Figure:**
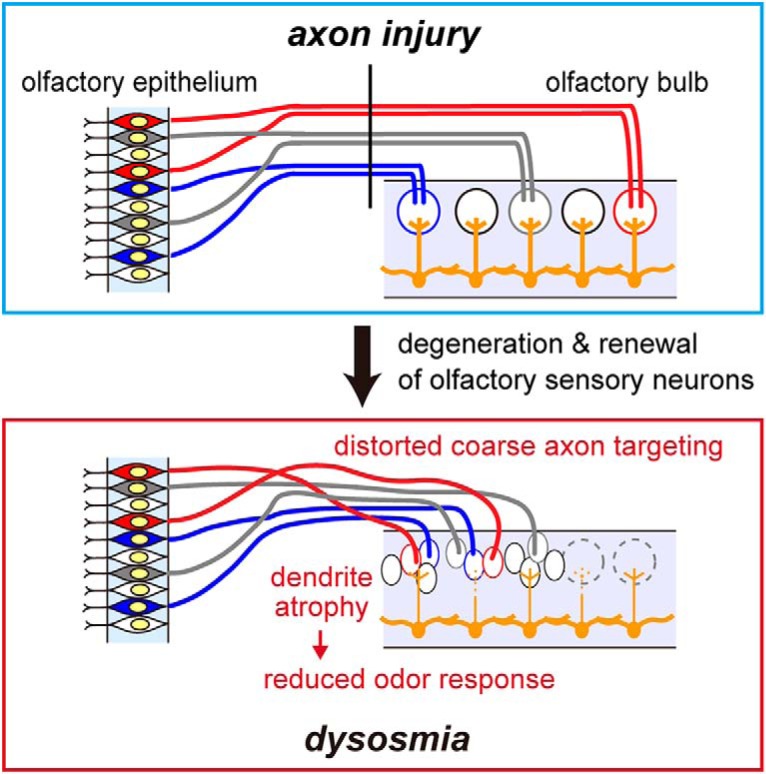

## Significance Statement

Olfactory sensory neurons (OSNs) have a unique ability to regenerate throughout the life of animals. Therefore, OSNs are newly generated and project to the olfactory bulb even after injury. However, it has been known that severe head trauma, which accompanies the transection of OSN axons, often leads to dysosmia (qualitative and quantitative changes in olfaction). Currently, there is no effective therapy for dysosmia patients. To gain mechanistic insights, we investigated the anatomical and functional changes of the olfactory system after OSN axon injury, and found defects in coarse OSN axon targeting and dendrite connectivity of mitral cells. Based on these findings, possible future strategies for dysosmia will be discussed.

## Introduction

The olfactory system is capable of detecting and discriminating a huge variety of airborne odorants. In addition, the olfactory system has an ability to judge the qualitative and social value of odors, perceiving them as pleasant and unpleasant. While the olfactory perception is generally stable throughout life, perceived olfactory quality is altered in an olfactory disorder known as dysosmia in humans. Dysosmia is often caused by a high-energy trauma to the head region (Yousem et al., 1996; Doty et al., 1997; Doty, 2009). Olfactory sensory neuron (OSN) axons are easily damaged by the shearing stress of the trauma, and dysosmia is typically recognized after a transient loss of olfaction. Post-traumatic dysosmia patients typically have reduced sensitivity to odors (hyposmia), with unpleasant olfactory perception of many kinds of odors (parosmia; Bonfils et al., 2005). The prognosis is bad, and currently there is no effective therapy to prevent or cure post-traumatic dysosmia (London et al., 2008). Although olfaction is not essential for life, it impairs the quality of life and can become a serious issue for patients. To move toward more effective therapies, it is important to understand the molecular and cellular processes underlying dysosmia.

The logic of olfactory perception has been well studied using rodent models (Axel, 2005; Buck, 2005; Mori and Sakano, 2011). Odors are detected by odorant receptors (ORs) expressed by OSNs in the olfactory epithelium (OE; Buck and Axel, 1991). There are ∼350 ORs in humans and ∼1000 ORs in mice. OSNs expressing the same type of OR converge their axons onto the same glomerulus in the olfactory bulb, forming a glomerular map. Thus, odor information is represented as a spatiotemporal activation pattern of ∼1000 sets of glomeruli in the olfactory bulb. Therefore, the glomerular map is important for odor recognition and discrimination. The glomerular map is initially formed during embryonic development (Imai et al., 2010), and, once established, this glomerular map is stable throughout the life of animals, even though OSNs are continuously replaced by newly generated OSNs as part of a turnover process (Schwob, 2002).

OSNs are also regenerated after OSN injury (Schwob, 2002). Glomerular map formation after the OSN injury has been studied in mice with chemical or physical lesions of OSNs. The outcome was, however, variable between the different lesion protocols. For example, a single intraperitoneal injection of methimazole results in the loss of most (though not all) mature OSNs, but they can re-establish the correct glomerular map without any defects (Blanco-Hernández et al., 2012). An injection of dichlobenil or the inhalation of methyl bromide causes more severe damage to OSNs, and regenerated OSN axons show mistargeting to the OB in the initial stage; however, in many cases, the glomerular map is largely recovered after a sufficient recovery period (Schwob et al., 1999; St John and Key, 2003; Carr et al., 2004; Cheung et al., 2014). In contrast, the glomerular map was poorly recovered when OSNs were injured by OSN axon transections (Costanzo, 2000; Christensen et al., 2001). It has been considered that the distorted glomerular map after diffuse OSN axon injury may underlie the altered olfactory perception in dysosmia patients. However, it has not been fully investigated how the glomerular map is disorganized after OSN injury. It has also remained unknown why newly generated OSN axons cannot re-establish the correct glomerular map when OSN axons were injured. Here we studied the cellular process that results in a distorted olfactory map after OSN injury, and found that anterior–posterior coarse targeting is disturbed. We also examined the OB circuitry and found that dendrite connectivity of mitral cells is reduced after the injury.

## Materials and Methods

### Mice

All the animal experiments were approved by the Institutional Animal Care and Use Committee of the RIKEN Kobe branch. Wild-type (C57BL/6N), *OMP-GFP* knock-in mice (RRID:IMSR_JAX:006667; Potter et al., 2001) and *MOR29A/29B* transgenic mice (Tsuboi et al., 2011) were used to study OSN projection; *Thy1-YFP* (line G, RRID:IMSR_JAX:014130; Feng et al., 2000) was used to label mitral cells in the OB; and *Thy1-GCaMP6f* (line GP5.11, RRID:IMSR_JAX:024339) was used for Ca^2+^ imaging of mitral cells *in vivo*. All of these mice were bred to C57BL/6N mice to maintain the mouse colony for this study. Male *OMP-GFP* mice were crossed with female ICR mice for *in utero* electroporation.

### *In utero* electroporation

*In utero* electroporation was performed as described previously (Saito, 2006). To label mitral cells, *in utero* electroporation was performed at embryonic day 12 (E12; Imamura and Greer, 2013). Two microliters of plasmid solution (pCAG-tdTomato, 1 μg/μl) was injected into the lateral ventricles, and electric pulses were delivered along the anterior–posterior axis with forceps-type electrodes (3 mm in diameter) and a CUY21EX electroporator (BEX).

### Axotomy

**Figure 1.**
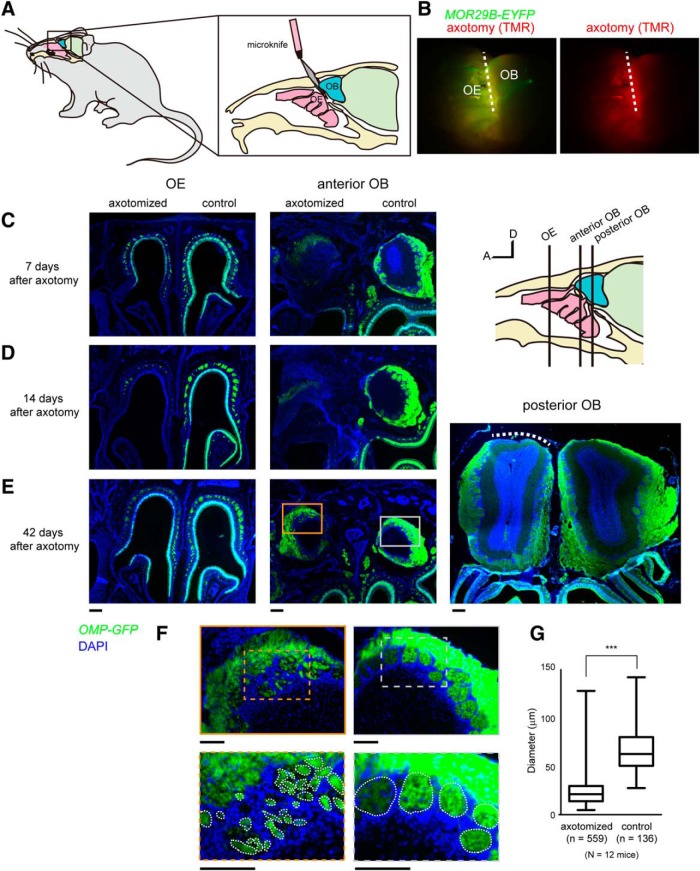
Mistargeting of OSN axons after OSN lesion and recovery. ***A***, Schematic representation of unilateral axotomy of dorsal-zone OSNs. Axon bundles of dorsal zone OSNs projecting to dorsolateral OB were transected near the cribriform plate. Only the right side was injured. ***B***, The micro knife was coated with TMR-dextran dye to visualize the incision site. *MOR29B-EYFP* mice were used. Axotomy was limited to dorsal zone OSNs (broken line). ***C***, OE and OB 7 d after the axotomy. ***D***, OE and OB 14 d after the axotomy. An almost complete OSN lesion in the anterodorsal OE and OB was observed in eight of eight mice at day 14. ***E***, Recovery of OSNs 42 d after axotomy. OSNs were regenerated, and the number of OSNs recovered to levels comparable to those of the controls on the noninjured side. However, OSN axons were scarce in the posterior part of the dorsal OB (right, white broken line). ***F***, High-magnification images of ***E***, middle, showing axotomized (orange) and control (gray) sides. OSN axons formed small glomerular-like structures in the anterior OB after axotomy. ***G***, Quantification of the diameter for glomerular-like structures in the control and axotomized sides. *OMP-GFP* mice were used in ***C****–****G***. ***p < 0.001 (Mann–Whitney *U* test). *n* = 559 (axotomy) and 136 (control) from 12 mice. A, Anterior; D, dorsal. Scale bars: ***C–E***, 200 μm; ***F***, 100 μm.

OSN axotomies were performed as described previously with some modifications (Costanzo, 2000). The 8- to 12-week-old mice (both male and female) were anesthetized with ketamine (80 μg/g body weight) and xylazine (7 μg/g), and then given dexamethasone (0.1 μg/g; Kyoritsu Seiyaku) and enrofloxacin (5 μg/g; Pfizer) by subcutaneous injection to prevent brain edema and infection. A dental drill was used to thin the nasal skull of the OE–OB border region (along the cribriform plate). OSN axons projecting to the dorsal OB were transected along the cribriform plate with a micro knife for ophthalmic surgery (2.8 mm, 45° bevel up, Beaver Xstar Slit Knife, Beaver-Visitec International). After axotomy, a coverslip was put onto the craniotomy with dental cement, and the skin flap was sutured. In most experiments, only OSN axons projecting to the right OB were transected. In some experiments, the left or both sides were axotomized. Mice were administered with daily subcutaneous injection of dexamethasone (0.1 μg/g; Kyoritsu Seiyaku) and enrofloxacin (5 μg/g; Pfizer) for 5 consecutive days. For recovery, mice were placed in single-housed cages and kept for 3–84 d. In Figure 1*B*, a micro knife was labeled with 5% tetramethylrhodamine (TMR)-dextran (10 kDa; catalog #D1868, Thermo Fisher).

### Immunohistochemistry of sections

Mice were deeply anesthetized with an overdose of Nembutal (Dainippon Sumitomo Pharma) administered by injection, followed by intracardiac perfusion with 4% paraformaldehyde (PFA) in PBS. Dissected OE and OB samples were further fixed in 4% PFA/PBS. Fixed OE–OB samples were decalcified with 0.5 m EDTA for 7 d, cryoprotected in 30% sucrose in PBS overnight, and then embedded in optimal cutting temperature compound. Cryostat sections (16 μm thick) were collected on glass slides and fixed with 4% PFA/PBS for 15 min. In the anti-Neuropilin 1 (Nrp1) immunostaining, sections were treated with 10 mm citrate buffer, pH 6.0, at 120°C, for 20 min for antigen retrieval. Blocking of sections was performed with 5% donkey normal serum. The primary antibodies used in this study were goat anti-Nrp1 [1:100; catalog #AF566, R&D Systems (RRID:AB_355445)], goat anti-Kirrel2 [1:100; catalog #AF2930, R&D Systems (RRID:AB_2130975)], and chicken anti-GFP [1:500; catalog #GFP-1010, Aves Labs (RRID:AB_2307313)]. Alexa Fluor 488-conjugated donkey anti-chicken IgY [catalog #703-545-155, Jackson ImmunoResearch (RRID:AB_2340375)] and Alexa Fluor 555-conjugated donkey anti-goat IgG [catalog #A-21432, Thermo Fisher (RRID:AB_2535853)] were used as secondary antibodies at 1:200 to 1:250. DAPI (Thermo Fisher) was used at 1:1000.

### Optical clearing and dendrite tracing

*Thy1-YFP* line G mice were used for dendrite-tracing experiments of mitral cells. Some experiments were performed with *OMP-GFP* mice with mitral cell labeling (tdTomato) by *in utero* electroporation. At 42 d after axotomy, OB was excised and fixed in 4% PFA/PBS, and sections (500 μm thick) were obtained using a microslicer (Dosaka). OB slices were cleared with SeeDB2G, as described previously (Ke et al., 2016). A confocal microscope (TCS SP8, Leica Microsystems) with a 20× objective lens [HC PL APO, 20× IMM CORR CS2; numerical aperture (NA) 0.75] was used to obtain three-dimensional images of enhanced yellow fluorescent protein (EYFP) and DAPI. Only the anterodorsal part of the OB was analyzed in sagittal slices, because this area is damaged by our axotomy protocol. Since the EYFP signals in this *Thy1-YFP* mouse line was heterogeneous among mitral cells, brightly labeled mitral cells (within the top 20%) were identified with ImageJ, and their primary dendrites were analyzed in an unbiased manner in this study. Only primary dendrites were traced in Figure 4 using Neurolucida software (MBF Bioscience). We only quantified neurons where whole primary dendrites were entirely contained within the acquired 3D volume. After the axotomy, mitral cells often possessed branches from the middle part of the primary dendrite. Tracing and quantification in Figure 4 were performed by one observer who was not blinded to the sample types. Only for the *Thy1-YFP* day 42 samples were all images reanalyzed by another observer who was blinded to the sample types, leading to the same conclusion (data not shown). In Figure 5, primary and lateral dendrites, including dendritic tufts were reconstructed using Neurolucida.

### *In vivo* two-photon Ca^2+^ imaging

We analyzed odor-evoked responses in the OB using the *Thy1-GCaMP6f* transgenic mice (line GP5.11), in which GCaMP6f is specifically expressed in mitral cells in the OB. Mice (8- to 16-week-old males; axotomized mice were 42 d postinjury) were anesthetized with ketamine/xylazine, and craniotomy was made onto the right OB. The skull overlying the dorsal OB was thinned with a dental drill and then removed with a fine forceps. Kwik-Sil (WPI) was applied on the OB, and a small coverslip (4 mm; MATSUNAMI) was placed onto the exposed OB. The coverslip was attached to the skull and surrounding thin wall with dental cement (GC UNIFAST II, Shofu). Butyric acid, acetophenone, and guaiacol were diluted at 1:100 in mineral oil. Vanillin was dissolved in dimethylsulfoxide at the saturated concentration. Anesthetized mice were exposed to odorants diluted further with air at 1:10 using an olfactometer (K.K. Matsumi Kogaku Keisoku). GCaMP6f was excited at 920 nm (InSight DS Dual, Spectra-Physics), and images were acquired with an upright two-photon laser-scanning microscope (FV1000MPE, Olympus) using a water-immersion 10× objective lens (UMPLFLN 10XW; NA, 0.30) and a GaAsP detector. In Figure 6*B*, the mean Δ*F*/*F*_0_ signals during the first 5 s of odor stimulation (single trial/animal) was averaged among samples after images were aligned based on the anterior and lateral edges of the OB. In Figure 6*C*, cumulative plots were made based on pixel-based Δ*F*/*F*_0_ signals for individual samples using MATLAB.

### Statistical analysis

Prism 5 and R were used for statistical analyses. Calcium-imaging data were analyzed using custom-written MATLAB scripts.

## Results

### Olfactory map disorganization by OSN axon transection

In humans, unmyelinated OSN axons connecting the OE and the OB are fragile, and dysosmia is often caused by damage to these axons following the head trauma. To recapitulate similar injuries in a mouse model, we performed a unilateral OSN axon transection (axotomy), as has been used in earlier studies (Costanzo, 2000; Christensen et al., 2001). To minimize unnecessary damage to the surrounding tissues, we made a small incision to the skull, and axon bundles projecting from the dorsal OE to the dorsal OB were transected with micro knives at the boundary between cribriform plate and OB (Fig. 1*A*,*B*). The ventral zone OSNs remained largely intact in our surgery. To minimize inflammatory responses, we administered dexamethasone for 5 consecutive days (Kobayashi and Costanzo, 2009). Degeneration and regeneration were first examined with *OMP-GFP* knock-in mice, in which all mature OSNs are labeled with GFP. The unilateral axonal transection caused a gradual retrograde degeneration of OSNs in the ipsilateral OE, resulting in complete elimination of mature OSNs within 14 d (Fig. 1*C*,*D*). Distal axons were also degenerated (Wallerian degeneration) by this point (Fig. 1*D*, right). Of eight axotomies performed by us, all showed complete loss of OSNs in the dorsal zone OE by day 14. OSNs were then gradually regenerated and recovered by day 42 after the injury (Fig. 1*E*). Of 12 *OMP-GFP* animals with unilateral axotomy, all demonstrated a recovery of OSNs at day 42.

We examined the overall axonal projection profiles of OSNs at 42 d postinjury. Axons of newly generated OSNs extended to the glomerular layer of the OB. However, these OSN axons formed small glomerular-like structures within the glomerular layer in the anterior part of the OB (Fig. 1*F*,*G*), which is consistent with earlier studies performed with chemical lesions (St John and Key, 2003; Cheung et al., 2014). Compared with the control, OSN axons tended to be scarcer in the posterior part of the dorsal OB (Fig. 1*E*, right).

### Anteroposterior coarse targeting is disturbed after axotomy

**Figure 2.**
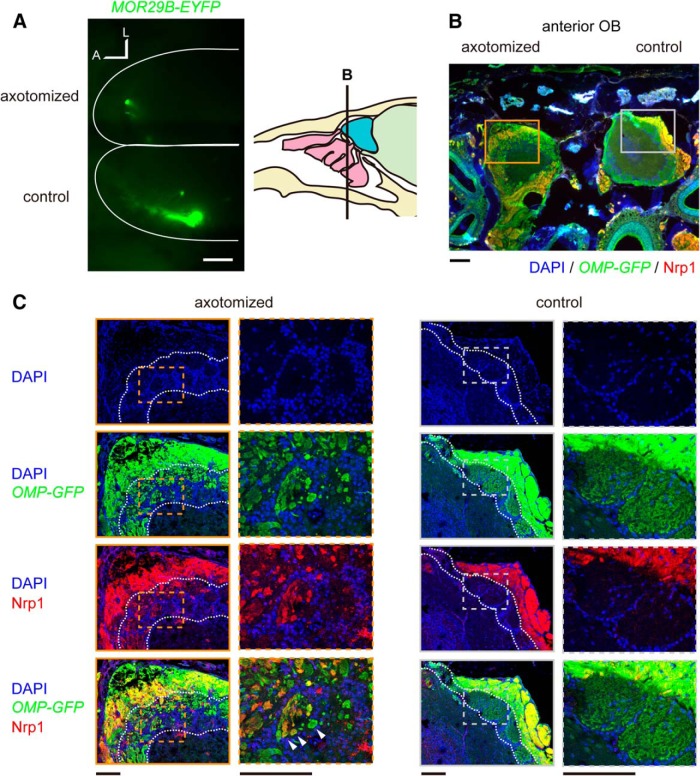
Anterior–posterior topography is disrupted after axotomy. ***A***, Defective axonal projection of OSNs expressing *MOR29B-EYFP*. On the control side, a major glomerulus for MOR29B was observed in the posterolateral area of the dorsal OB. However, MOR29B-expressing OSNs projected to the anterior and/or medial region of the OB after OSN axon injury (42 d recovery; in six of six animals analyzed). A, Anterior; L, lateral. ***B***, Mistargeting of Nrp1-positive OSN axons to the anterior OB. ***C***, High-magnification confocal images of boxed areas in ***B***. On the control side, Nrp1-positive axons were sorted to the superficial layer of the olfactory nerve layer and projected to the posterior OB. However, on the axotomized side, Nrp1-positive axons were found in the deeper portions of the olfactory nerve layer, and invaded glomeruli in the anterior OB. Nrp1-positive and Nrp1-negative axons formed small glomerular-like structures within glomeruli in the anterior OB (arrowheads). Glomerular layer is demarked by dotted lines. An OMP-GFP mouse was analyzed. Note that Nrp1-positive and Nrp1-negative OSN axons formed distinct glomerular-like structures within glomeruli in the high-magnification images. Scale bars: ***A***, 500 μm; ***B***, 200 μm; ***C***, 100 μm.

Next, we examined the axonal projection profiles for OSNs expressing transgenic *MOR29B* (Tsuboi et al., 2011). *MOR29B* (*Olfr1511*) is a class II OR gene expressed in the dorsal zone of the OE. On the control side, *MOR29B*-expressing OSN axons projected to the dorsolateral part of the OB; however, they did not project to the original location in the OB, and instead formed smaller glomeruli in the anterior (*n* = 4/6; Fig. 2*A*) or more medial (*n* = 2/6) part of the OB after axotomy.

During development, the anterior–posterior topography of the OB is, at least partly, regulated by Nrp1 and semaphorin 3a (Sema3A) signaling (Schwarting et al., 2000; Taniguchi et al., 2003; Imai et al., 2009). Nrp1 expression levels in OSNs determine the anterior–posterior positioning of glomeruli, with low Nrp1 to anterior OB and high Nrp1 to posterior OB. Sema3A is expressed not only in the anterior OB, but also by intermediate cues en route (ensheathing glia) and OSN axons projecting to anterior OB. As a result, Sema3A–Nrp1 signaling regulates both axon–axon and axon–target interactions to guide Nrp1-high axons to the posterior OB. Here we examined axonal projection of Nrp1-expressing OSNs. In the control OB, Nrp1-high axons are sorted to the superficial layer of the olfactory nerve layer and projected to the posterior OB. In contrast, on the axotomized side, Nrp1-high axons were often ectopically located in the deep layer of the nerve and projected to the glomeruli in the anterior part of the OB (*n* = 12/12; Fig. 2*B*,*C*). Thus, the poor convergence of OSN axons after axotomy (Costanzo, 2000) is due to defects in coarse axon targeting (Imai et al., 2009), rather than defects in local axon sorting (Yu et al., 2004; Serizawa et al., 2006).

### Heterogeneous OSN axons form segregated glomerular-like structures within a glomerulus

**Figure 3.**
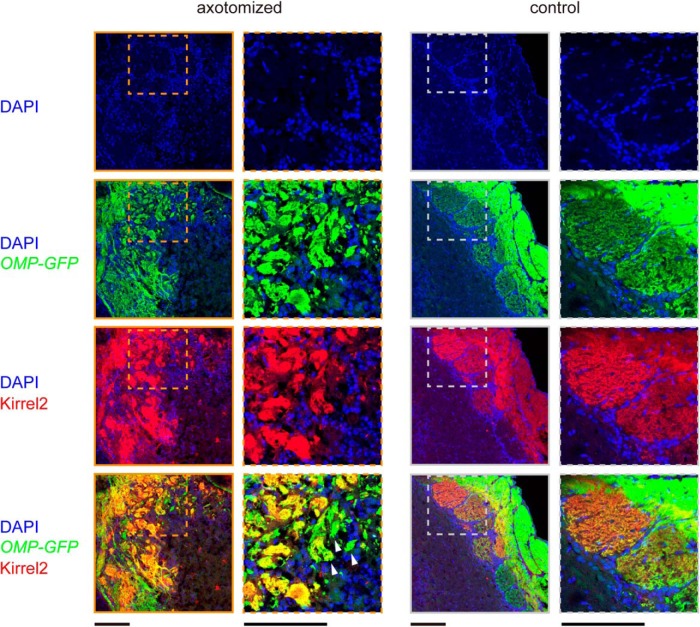
Heterogeneous OSN axons converge onto a glomerulus. OB sections stained with anti-GFP (*OMP-GFP*) and anti-Kirrel2 antibodies were imaged with confocal microscopy. DAPI staining shows the distribution of periglomerular cells surrounding glomeruli. The layout of periglomerular cells remains unchanged. The glomerular layer is demarked by dotted lines. The anterior part of the dorsal OB is shown. In the control OB, Kirrel2 immunoreactivity was glomerulus specific and homogeneous within a glomerulus. However, on the axotomized side, distinct glomerular-like structures with differential Kirrel2 levels were found within each glomerulus (arrowheads). Similar results were obtained with 17 experiments. Scale bars, 100 μm.

In the anterior part of the OB, Nrp1-high and Nrp1-low OSN axons were often intermingled within a glomerulus (Fig. 2*C*). To further examine the heterogeneity of OSN axons within a glomerulus, we also analyzed the localization of Kirrel2, another OR-regulated cell recognition molecule that is involved in local axon sorting (Serizawa et al., 2006). In the control OB, each glomerulus showed a unique expression level of Kirrel2. Within each glomerulus, the Kirrel2 level was almost homogeneous, reflecting the convergence of homogeneous OSN axons expressing a common OR. After the axotomy, the layout of periglomerular cells remained similar to the control, forming glomeruli 50–80 μm in diameter (Fig. 3). On the axotomized side, however, Kirrel2-high and Kirrel2-low OSN axons were often found in the same glomerulus, forming discrete glomerular-like structures within a glomerulus (Fig. 3). High-magnification images showed that Kirrel2-high and Kirrel2-low axons were still segregated within a glomerulus, indicating that local axon-sorting mechanisms (Serizawa et al., 2006) remain intact even after axotomy. These results together demonstrate that heterogeneous OSN axons project to a common glomerulus after axotomy.

### Connectivity of mitral cell dendrites to glomeruli was reduced after axotomy

**Figure 4.**
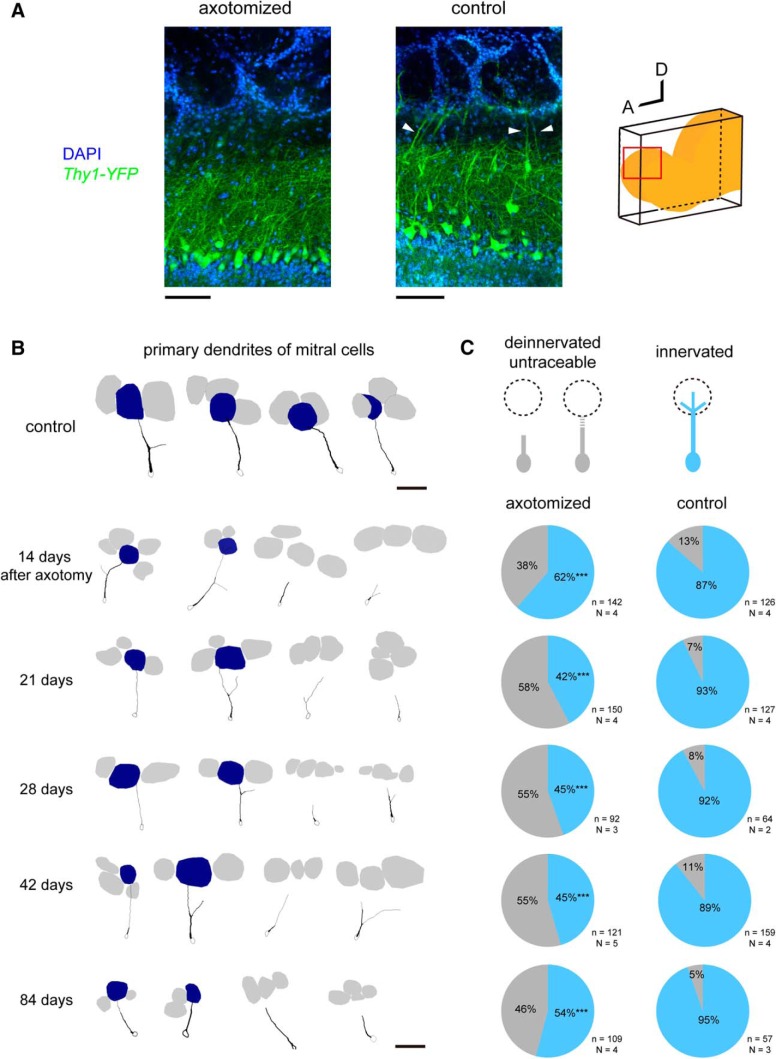
Connectivity of mitral cell dendrites is reduced after axotomy. ***A***, Stacked confocal images (20 μm thick) of OB slices from *Thy1-YFP* (G line) mice. Mitral cells are labeled with YFP in this mouse line. Sagittal OB slices were cleared with SeeDB2G, and anterodorsal part (red) was analyzed with confocal microscopy. Note that control OBs show thick primary dendrites extending to the glomerular layer (white arrowheads), whereas only faint signals were seen in the outer external plexiform layer under axotomized conditions. For unbiased quantification, brightly labeled mitral cells (top 20%) were traced for quantification. ***B***, Representative Neurolucida tracing of mitral cell primary dendrites in the control and axotomized OBs. Note that only primary dendrites are shown. Primary dendrites of mitral cells partially deinnervated from glomeruli in the axotomized side. Innervated glomeruli are shown in blue, and other glomeruli are shown in gray. ***C***, Quantification of primary dendrite innervation. Mitral cell dendrites were often thinned and became untraceable at 21 d after axotomy. The partial deinnervation of primary dendrites persisted for at least 84 d after axotomy. The numbers of mitral cells (*n*) and animals (*N*) are indicated. ***p < 0.001 (χ^2^ test for mitral cells with innervating dendrites, compared with the control side). Scale bars, 100 μm.

After recovering from axotomy, newly generated OSNs project to the OB (Costanzo, 2000; Christensen et al., 2001); however, it has not been fully examined whether these OSNs form functional connections in the OB. It is unknown whether mitral cell dendrites remain intact after the deafferentation of OSNs. To examine the connectivity of mitral cell dendrites after axotomy, we used *Thy1-YFP* (line G) mice (Feng et al., 2000), in which mitral cells are brightly labeled with EYFP. The OB slices were cleared with SeeDB2G (Ke et al., 2016), and 3D fluorescence images were acquired with confocal microscopy. To ensure tracing accuracy, primary dendrites of brightly labeled mitral cells (top 20th percentile) were traced in an unbiased manner from the somata to the tip of primary dendrites. We analyzed the anterodorsal part of the OB, where OSN axons deinnervated and reinnervated. In the control OB, most mitral cells (80–90%) extended thick primary dendrites to the glomerular layer of the OB. However, 21 d after axotomy, thick primary dendrites were rarely observed in this OB region; in our light microscopy-based tracing, ∼50% of mitral cells failed to show dendrite connection to the glomerular layer, suggesting a partial deinnervation or reduced dendrite thickness (Fig. 4*A*).

**Figure 5.**
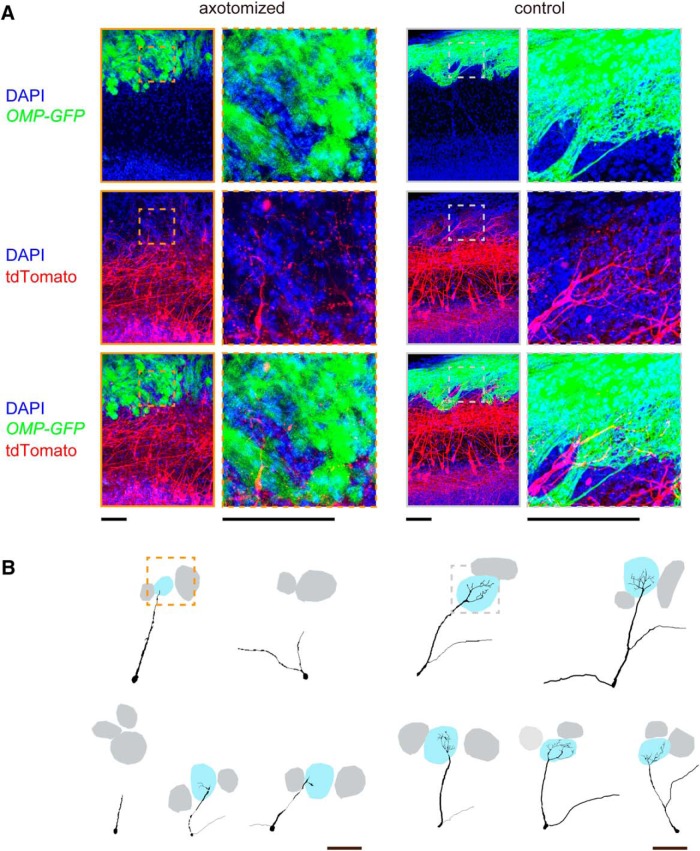
Atrophy of dendritic tufts in mitral cells. ***A***, Mitral/tufted cells were labeled with tdTomato using *in utero* electroporation at E12. *OMP-GFP* mice were used to visualize OSNs. Axotomy was performed at postnatal day 56, and mitral cell morphology was analyzed 42 d after axotomy. The *z*-stacked confocal images (52 μm thick) are shown. High-magnification images show the tufted structures within a glomerulus in the control, but not after axotomy. ***B***, Representative Neurolucida tracing of mitral cell dendrites, including tufted structures within glomeruli. We show five representative neurons, including the ones shown in ***A***. Boxed areas are shown in high-magnification images in ***A***. Scale bars, 100 μm.

To more clearly visualize morphological changes in the dendritic tufts, we performed fluorescence labeling of mitral cells with *in utero* electroporation (Saito, 2006; Imamura and Greer, 2013). In this labeling, mitral/tufted cells were more sparsely and brightly labeled, facilitating the visualization of dendritic tufts. In the control OB, highly ramified dendritic tufts were clearly visualized in the glomerular layer. In contrast, mitral cells rarely innervated the glomerular layer and poorly ramified within glomeruli after axotomy (Fig. 5). It should be noted, however, that with light microscopy it is technically difficult to conclude whether some of the mitral cells have completely lost connectivity to glomeruli or whether they still maintain very thin processes; at the minimum, our results indicate that primary dendrites become thinner and are weakened in a substantial population of mitral cells after axotomy. The defective dendrite innervation did not fully recover even at day 42 or 84, when the projection of OSN axons has fully recovered in the anterior part of the OB (Fig. 4). We also obtained consistent results using *in utero* electroporation (data not shown).

### Odor responses in the OB after OSN lesions and subsequent regeneration

**Figure 6.**
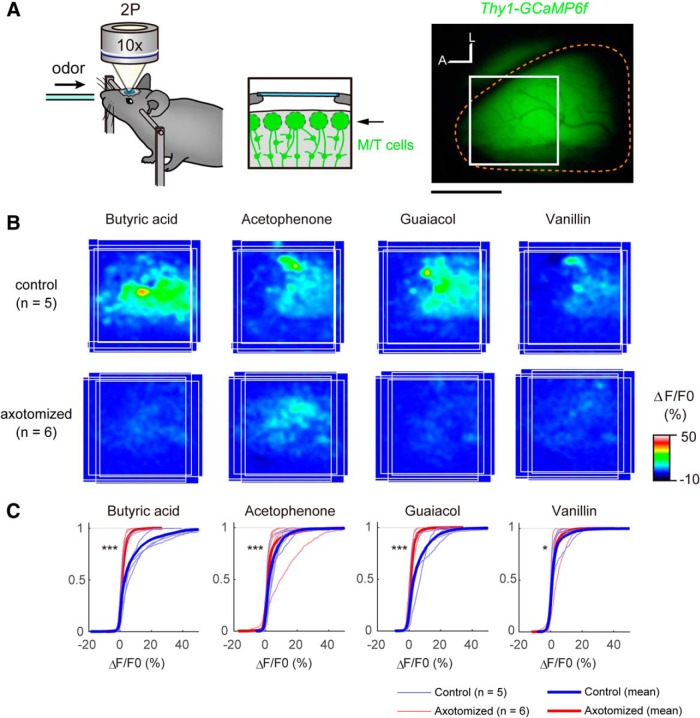
Odor responses in mitral cells are reduced after axotomy. ***A***, Two-photon Ca^2+^ imaging of the olfactory bulb using *Thy1-GCaMP6f* mice (GP5.11). In this mouse line, GCaMP6f is specifically expressed in mitral cells. The glomerular layer in the anterodorsal part of the OB (boxed area) was imaged using two-photon microscopy. Right OBs (demarcated by orange broken lines) were analyzed. A, Anterior; L, lateral. ***B***, The odor map after axotomy was altered (day 42). Odor-evoked responses in the OB were analyzed by two-photon Ca^2+^ imaging. Average activation maps are shown. In the control OB, butyric acid activates the anteromedial part of the dorsal OB (class I), whereas acetophenone, guaiacol, and vanillin activated more of the posterolateral area (class II). However, the response amplitude was much reduced, and localization was not clear after the recovery after axotomy. ***C***, Cumulative curves for odor-evoked responses (pixel-based Δ*F*/*F*_0_ during the first 5 s of odor exposure). After recovery from axotomy, odor-evoked responses in the OB were much reduced. *p < 0.05, ***p < 0.001 (Kolmogorov–Smirnov test). Scale bars, 1 mm.

To examine the functional consequences of aberrant regeneration after axotomy, we performed Ca^2+^ imaging of the OB using the *Thy1-GCaMP6f* transgenic mouse (line GP5.11), in which a genetically encoded Ca^2+^ indicator, GCaMP6f, is specifically expressed in mitral cells in the OB (Dana et al., 2014). We analyzed odor-evoked responses in the glomerular layer. It should be noted that nearly half of the mitral cells still maintain connections to glomeruli, even after axotomy (Fig. 4). In mice, dorsal OB can be divided into three distinct domains by genetic and/or molecular markers as well as by odor response signatures, as follows: D_I_ for OSNs expressing class I ORs; D_II_ for class II ORs; and D_III_ for Tarr ORs (Kobayakawa et al., 2007; Bozza et al., 2009; Pacifico et al., 2012). We used one odor that specifically activates the D_I_ domain (Butyric acid) and three odors that specifically activate the D_II_ domain (acetophenone, vanillin, and Guaiacol; Bozza et al., 2009), and analyzed the anterodorsal part of the OB spanning these two domains. In the control OB, responses to these odors were robust, and activation domains for D_I_ and D_II_ odors were largely segregated. In contrast, on the axotomized side, the odor-evoked responses were much reduced, as demonstrated by the cumulative plots of Ca^2+^ responses (Δ*F*/*F*_0_ of GCaMP signals; Fig. 6). Thus, the odor-evoked responses are dramatically changed after the axotomy, even though newly generated OSNs project axons to the OB.

## Discussion

### Qualitative changes of olfaction may be due to disrupted domain organization of the glomerular map

To recapitulate the post-traumatic dysosmia, an earlier study performed axotomy for *P2-taulacZ* knock-in mice in which an odorant receptor gene *P2* was genetically labeled (Costanzo, 2000). OSNs expressing the *P2* gene project their axons to the anterior part of the OB. While this study demonstrated dispersed axonal projection after OSN regeneration, the overall changes in the glomerular map have not been fully described. Therefore, it has been unknown whether changes were due to defects in coarse axon targeting or to local axon sorting (Imai et al., 2010). In the present study, we performed axotomy for dorsal zone OSNs and analyzed axonal projection of *MOR29B* OSNs. We found that *MOR29B* OSN axons were often mistargeted to more anterior or medial OB regions after the axotomy (Fig. 2*A*). The distorted anterior–posterior topography was also confirmed by anti-Nrp1 immunostaining of OB sections (Fig. 2*B*,*C*). In the anterior OB, multiple heterogeneous glomeruli were formed within a glomerulus (Fig. 3). Since different types of odors are represented by different domains of the OB (Mori et al., 2006; Bozza et al., 2009), the defective coarse axon targeting may underlie qualitative changes in odor perception after traumatic OSN axon injury. Because we only transected dorsal-zone OSN axons in our analysis, we do not exclude the possibility that dorsal–ventral topography of the OB may also be disrupted when both dorsal-zone and ventral-zone axons are injured (Costanzo, 2000).

### Reduced olfactory sensitivity after OSN axon injury

It has been demonstrated that the regenerated OSNs after injury are functional and respond to odors (Cheung et al., 2014). However, the anatomy and odor responses in mitral cells have not been investigated so far. In the present study, we found that the connectivity of mitral cell primary dendrites is reduced after the OSN injury. It is known that the tufted structure of the mitral cell primary dendrites is normally stable in adults (Mizrahi and Katz, 2003); however, our results indicate that OSN axons are required to stably maintain the dendrite morphology of mitral cells (Figs. 4, 5). Previously, atrophy of mitral cell dendrites has been reported for mice where OSN axons were eliminated from early developmental stages, before the formation of mature tufted structures (Leo and Brunjes, 2003; Kobayakawa et al., 2007). Our present study demonstrated that the dendritic retraction of mitral cells can occur in the adult, even after the maturation of dendrites. Furthermore, once retracted, it is difficult to re-establish the connectivity even after OSN projection recovered. It will be interesting to study in the future how OSN-derived signals control maturation and/or maintenance of mitral cell dendrites in the OB.

Consistent with the anatomical evidence, we also found that odor-evoked responses in mitral cells are much reduced (Fig. 6). Thus, the reduced connectivity of mitral cell dendrites may be a cause for the reduced sensitivity after OSN axon injury (hyposmia; Bonfils et al., 2005). It is known that the remodeling of mitral cell dendrites is active during an early postnatal period (Malun and Brunjes, 1996). In contrast, we could not find evidence for dendrite re-innervation/growth even at 84 d after axotomy, suggesting that the plasticity and growth of mitral cells are limited in the adult OB. It is possible that there is a critical period for mitral/tufted cell dendrite remodeling, as has been shown in other sensory systems (Hensch, 2005). In the OB, OR-specific convergence of OSN axons is the basis of odor discrimination and odor information processing in the OB (Imai, 2014). Therefore, the convergence of multiple heterogeneous OSN axons to a glomerulus (Figs. 2, 3) may also have been a cause of reduced responses in mitral cells.

### OSN axons are required to maintain the glomerular map and its connectivity with mitral cell dendrites in the adult

**Figure 7.**
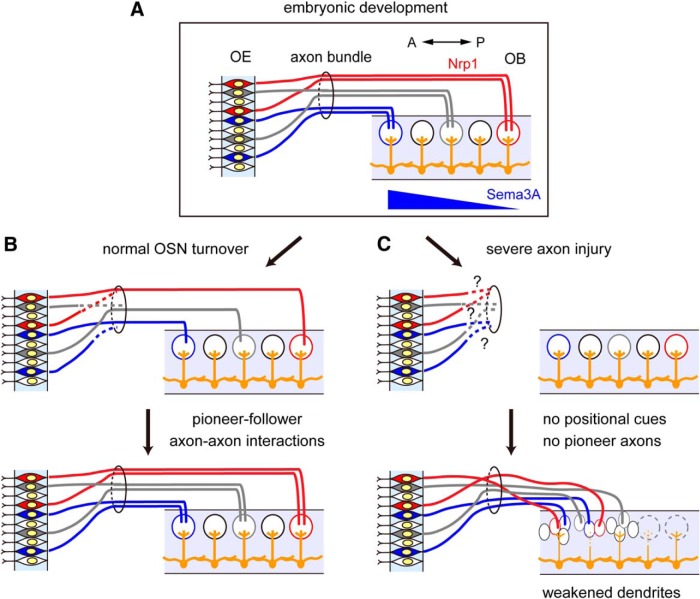
Schematic representation of defective circuit recovery after axotomy. ***A***, Embryonic development of the olfactory map. Target cues (e.g., Sema3A) guide axons to correct positions in the OB. A, Anterior; P, posterior. ***B***, Normal OSN turnover process. We assume that axon–axon interactions (pioneer–follower interactions) are important to maintain the topographic olfactory map in the normal turnover process. ***C***, Our proposed model for defective OSN projection and partial dendrite deinnervation after axotomy. Without positional cues and axon–axon interactions, regenerated OSN axons cannot find correct targets in the OB after axotomy. Reduced connectivity of primary dendrites may lead to reduced odor responses in mitral cells.

Why is the regeneration process after OSN axon damage different from the normal OSN turnover process? We found that Nrp1-positive OSN axons are often mistargeted to the anterior OB after axotomy (Fig. 2). This may indicate that the target cues are no longer functioning in the adult (Fig. 7*A*). In fact, one of the genes for positional cues, *Sema3a*, is localized at the anterior OB only during embryonic stages (Schwarting et al., 2000). Our results are also consistent with those of recent studies using transgenic manipulations, showing a critical period for correct olfactory map formation (Ma et al., 2014; Tsai and Barnea, 2014). In the adult, regenerating OSNs depend more on axon–axon interactions in the adult (Fig. 7*B*; Imai and Sakano, 2011). Consistent with this idea, an earlier chemical lesion study with methimazole did not entirely eliminate OSN axons, and also did not lead to olfactory map distortion (Blanco-Hernández et al., 2012). In contrast, OSN axons were almost completely degenerated by axotomy. Without scaffolds for follower OSN axons, the coarse mapping of OSN axons may be disturbed after traumatic OSN axon injury (Fig. 7*C*). In contrast, local axon-sorting mechanisms remained functioning; as a result, Kirrel2-high and Kirrel2-low axons were nicely segregated to form discrete glomerular-like structures within glomeruli (Fig. 3). In the present study, we also found that primary dendrites of mitral cells partially deinnervated from glomeruli after OSN injury. This result also indicates the requirement of OSN axons in maintaining the mitral cell connectivity and OB circuits in the adult. Our results in the olfactory system also provide insights for regenerative medicine in the nervous system: the regeneration and regrowth of axons are not sufficient for functional recovery.

What kind of therapeutic approaches could minimize these problems after post-traumatic OSN injury? Since the physical existence of OSN axons seems to be important to maintain olfactory map topography and its connectivity, one possible approach is to minimize the degeneration of OSN axons after injury. If we can delay the degeneration of injured OSN axons, the regenerated OSN axons may catch up and form correct circuitry in the OB. It is known that the degeneration of distal axons after axonal injury is a result of an active degeneration mechanism, known as Wallerian degeneration (Conforti et al., 2014). In several types of neurons, ectopic expression of WldS (a fusion protein between Nmnat1 and Ube4b) or Nmnat1 are shown to delay Wallerian degeneration up to a few weeks. Unfortunately, however, our experiments with *WldS* mice did not show any delays in degeneration after OSN axotomy (data not shown), suggesting alternative and/or additional mechanisms in OSNs. Identifying the active axonal degeneration mechanisms in OSNs may open a new therapeutic possibility for post-traumatic dysosmia.

## Synthesis

Reviewing Editor: Michael Fainzilber, Weizmann Institute of Science

Decisions are customarily a result of the Reviewing Editor and the peer reviewers coming together and discussing their recommendations until a consensus is reached. When revisions are invited, a fact-based synthesis statement explaining their decision and outlining what is needed to prepare a revision will be listed below. The following reviewer(s) agreed to reveal their identity: Alain Trembleau

Both reviewers agree that this work is overall well done and adds significant knowledge to the previous literature. Nonetheless the following points should be addressed before a final decision is made regarding this manuscript:

Main point:

It is difficult for the reader, from the pictures provided, to assess the presence/absence (and qualitative differences if there are some, in terms of branching for example) of M/T cell dendritic arbor within glomeruli. Fig. 4A (Thy1-YFP) shows individual glomeruli nicely delineated by periglomerular cells, but one hardly see any intraglomerular dendritic arbor within the glomeruli. Fig. 5 (electroplated Tomato) shows better M/T cell dendrites and their arbor, but these arbors are located in areas homogeneously and densely packed with DAPI-stained nuclei (belonging presumably to periglomerular cells) where no clear glomeruli can be delineated. Whereas these differences (visible or not visible glomeruli) may be partly due to the fact that the thickness of the sections in Fig. 5 is higher, we still expect to see some glomeruli given the fact that their diameter is about 50-80 microns, while the thickness of the sections is 52 microns.

High mag and/or camera lucida drawings documenting M/T cell dendrites on Fig 4A would be useful, and other illustrations allowing the visualization of the dendritic arbors within glomeruli should be provided.

Additional points :

- In the "Significant statement", the sentence "OSNs can recover and regrow to the olfactory bulb even after injury" is mis-leading. OSNs do not survive axon trans-section, they do not recover : they just die. Regrowing axons do not come from injured OSNs, but from newborn neurons in the OE, thanks to the adult neurogenesis occurring in this tissue. Likewise in the text (i.e. lines 227, 228), "Regenerated OSN axons" is somehow ambiguous ; I would rather prefer "new OSN axons".

- Figure 1B would gain in clarity for non specialist readers if some labels/orientation information were aded onto the picture.

- Line 224 : I do not agree with the statement "... recovered to near-normal levels by day 42...". It is clear from Fig. 1E that in the OE, as well as in the OB, regeneration of new OSNs and of their axonal projections is not complete and quite far from normal. Quantification of OSN numbers would probably show significant difference.

- Line 249 : "OSN nerve layer" should be changed in "olfactory nerve layer"

- Line 252 : Fig. 2C illustrates the point much better that Fig. 2B as it is written.

- Line 258 : Fig. 2A does not illustrate this point. Did they authors mean Fig. 2C?

- Line 265 : I did not find the yellow arrowheads in Fig. 3

- Line 353-354 : "OSN axons were denervated" should be changed in "OB was denervated" or "OSN axons were eliminated"
